# Extracellular Vesicles in Chronic Lymphocytic Leukemia: Tumor Microenvironment Messengers as a Basis for New Targeted Therapies?

**DOI:** 10.3390/cancers15082307

**Published:** 2023-04-14

**Authors:** Kenza Dubois, Mariana Tannoury, Brigitte Bauvois, Santos A. Susin, Delphine Garnier

**Affiliations:** Sorbonne Université, Université Paris Cité, Inserm, Centre de Recherche des Cordeliers, Cell Death and Drug Resistance in Lymphoproliferative Disorders Team, F-75006 Paris, France; kenza.dubois@universite-paris-saclay.fr (K.D.); mariana.tannoury@inserm.fr (M.T.); brigitte.bauvois@sorbonne-universite.fr (B.B.); santos.susin@sorbonne-universite.fr (S.A.S.)

**Keywords:** chronic lymphocytic leukemia, extracellular vesicles, exosomes, tumor microenvironment

## Abstract

**Simple Summary:**

Chronic lymphocytic leukemia (CLL) is characterized by the accumulation of abnormal B lymphocytes in the peripheral components of the immune system. Despite the development of new therapies for CLL, drug resistance and disease relapse still occur. In the bone marrow and secondary lymphoid tissues, the trafficking, survival, and proliferation of leukemic B cells are regulated by interactions with the microenvironment (via cell-extracellular matrix interactions, cell-cell contacts, and the exchange of soluble factors) and contribute to treatment resistance. Here, we review the biology of the extracellular vesicles released into this microenvironment with cross-talk between neoplastic B cells and neighboring or remote target cells. A better understanding of the extracellular vesicles’ role in CLL progression and drug resistance might open up opportunities for the development of novel therapeutics that target the pro-survival dialogue between tumor cells and the tumor microenvironment.

**Abstract:**

In addition to intrinsic genomic and nongenomic alterations, tumor progression is also dependent on the tumor microenvironment (TME, mainly composed of the extracellular matrix (ECM), secreted factors, and bystander immune and stromal cells). In chronic lymphocytic leukemia (CLL), B cells have a defect in cell death; contact with the TME in secondary lymphoid organs dramatically increases the B cells’ survival via the activation of various molecular pathways, including the B cell receptor and CD40 signaling. Conversely, CLL cells increase the permissiveness of the TME by inducing changes in the ECM, secreted factors, and bystander cells. Recently, the extracellular vesicles (EVs) released into the TME have emerged as key arbiters of cross-talk with tumor cells. The EVs’ cargo can contain various bioactive substances (including metabolites, proteins, RNA, and DNA); upon delivery to target cells, these substances can induce intracellular signaling and drive tumor progression. Here, we review recent research on the biology of EVs in CLL. EVs have diagnostic/prognostic significance and clearly influence the clinical outcome of CLL; hence, from the perspective of blocking CLL-TME interactions, EVs are therapeutic targets. The identification of novel EV inhibitors might pave the way to the development of novel combination treatments for CLL and the optimization of currently available treatments (including immunotherapy).

## 1. Introduction

### 1.1. Chronic Lymphocytic Leukemia

Chronic lymphocytic leukemia (CLL) is the most common leukemia in Western countries and is characterized by the accumulation of malignant CD5+ CD19+ B cells with a defect in apoptosis and a very low proliferation rate [1]. The disease is heterogeneous in both molecular and clinical terms. Some cases of CLL are indolent and do not require treatment, whereas others are very aggressive. Clinically, patients are typically classified according to the Rai [2] and Binet [3] staging systems, which are based on blood cell counts and other variables. On the molecular and genetic levels, several chromosomal alterations are indicative of a poor prognosis; these include the deletion of the short arm of chromosome 17 (del17p), the long arm of chromosome 11 (del11q), trisomy 12, del13q, *TP53* point mutations, and an unmutated immunoglobulin heavy chain region (*UM-IGHV*) [1].

The CLL B cells’ proliferation rate is high when the cells reside in lymphoid organs such as the bone marrow (BM), lymph nodes (LNs), and spleen, but their proliferation rate is low in the peripheral blood (PB) [4,5]. The survival and proliferation of CLL cells are highly dependent on the B cell receptor (BCR) pathway. Once activated by external antigens or self-antigens, the BCR recruits spleen tyrosine kinases (SYKs) and Lck/Yes novel tyrosine kinase (LYN) and thus induces a phosphorylation cascade that activates Bruton’s tyrosine kinase (BTK), phosphoinositide 3-kinase (PI3K), protein kinase C, and Ras-dependent extracellular signal-regulated kinase [4]. The end result is the upregulation of nuclear factor kappa B (NF-κB) and CLL cell survival and proliferation [4]. As a result of genetic lesions or changes in signal transduction, anti-apoptotic proteins from the B-cell lymphoma/leukemia 2 (BCL2) family (Bcl-2, Mcl-1, and Bcl-xL) are overexpressed in CLL and are associated with a poor prognosis [6]. High expression levels of several tumor-associated antigens (including CD20, CD19, and CD22) in CLL are correlated with a high proliferation rate and disease progression and modulate BCR-dependent and BCR-independent proliferation/survival signals [6].

Current treatments for CLL include broad-spectrum cytostatic agents, targeted therapies, and combinations thereof [1,6,7]. The cytostatic agents include chlorambucil, fludarabine, and bendamustine. Novel inhibitors of BCR-associated kinases (i.e., the BTK inhibitors ibrutinib, acalabrutinib, zanubrutinib, and pirtobrutinib and the PI3K inhibitor idelalisib) have been approved in the USA and Europe for relapsed CLL or first-line treatment of a CLL patient bearing a *TP53* abnormality [6,7]. First-line treatment with idelalisib has been withdrawn as a result of adverse drug events (including hepatoxicity) but is still used in combination with other drugs [6,7].

Another treatment option is venetoclax, a BH3 mimetic that inhibits Bcl-2′s survival function and has been approved for the treatment of relapsed CLL patients—including those bearing a del17p [7]. Lastly, two anti-CD20 agents are currently approved by the US Food and Drug Administration for the treatment of CLL: rituximab is particularly effective when combined with chemotherapy, and obinutuzumab is less toxic than rituximab [1,6,7].

However, the treatment of CLL remains a challenge in the clinic because a significant proportion of patients are either refractory to the available first-line treatment or relapse after treatment. Although ibrutinib is one of the most frequent first-line treatments, continuous treatment leads to relapse and the acquisition of resistance; this is mainly associated with mutations in BTK or phospholipase C gamma 2 [6,8,9]. A similar process is observed with venetoclax, resistance to which is correlated with the acquisition of a mutation in *BCL2* [9]. Novel therapies are therefore needed to overcome resistance to existing drugs, and the identification of new therapeutic targets in CLL is of general interest.

### 1.2. The CLL Tumor Microenvironment

In addition to intrinsic genomic alterations (including oncogenic mutations and chromosomal rearrangements), tumor progression and drug resistance are greatly influenced by the interactions between CLL cells and the surrounding tumor microenvironment (TME) [10,11,12]. The normal cells in the TME (mainly immune cells (T cells and monocyte-derived cells) and stromal cells (including mesenchymal stromal cells (MSCs), endothelial cells, and fibroblasts)) form a niche in which CLL cells are protected from apoptosis, as evidenced by the high proportion of cells that die spontaneously when CLL cells are separated from the TME and cultured in vitro [13]. CLL cell survival is promoted by tumor-associated macrophages known as nurse-like cells through the secretion of a proliferation-inducing ligand (APRIL), a B-cell activating factor (BAFF), the C-X-C motif chemokine CXCL12 (also known as stromal cell-derived factor 1 (SDF-1)), and soluble CD14 or the lymphocyte function-associated antigen 3 (LFA-3)/CD2 interaction [12]. Similarly, endothelial cells are activated by CLL cells and then secrete BAFF and APRIL which, in turn, promote CLL cell survival [14]. MSCs protect tumor cells against spontaneous and drug-induced apoptosis by activating BCR and changing the expression of antiapoptotic molecules, SDF-1, and vascular endothelial growth factor (VEGF) [10,15]. Interestingly, stromal cells (such as MSCs and fibroblasts) in contact with the tumor can transdifferentiate into “activated” cancer-associated fibroblasts (CAFs). The CAFs further contribute to tumor progression through a phenotypic shift that notably regulates the formation of metastases, the production of the extracellular matrix, and the release of cytokines and growth factors [16].

One of the best illustrations of the TME’s influence on CLL biology is the difference in proliferation between quiescent CLL cells in PB and actively proliferating CLL cells in lymphoid secondary organs (especially LNs) [4]. Activated CD4 T cells can stimulate CLL cells and induce their proliferation through CD40 ligand (CD40L)-mediated cell–cell interactions [17,18].

The fact that cell metabolism reprogramming is clearly involved in tumor progression and dissemination has prompted growing interest in cancer research and drug development over the last decade [19]. In the particular context of CLL, the results of several studies have demonstrated that the TME can increase tumor cell survival by modulating tumor cells’ mitochondrial oxidative phosphorylation and nucleotide synthesis [20], favoring protection against oxidative stress by promoting glutathione synthesis [21] and causing a glycolytic switch through Notch-c-Myc signaling [22]. The increase in the glycolytic phenotype of CLL cells in LNs is BCR-dependent, although a specific subpopulation of CLL cells with a del17p (often correlated with *TP53* mutation) appears to spontaneous display this metabolic characteristic [23]. Chen et al. recently showed that the LN microenvironment induces dramatic metabolic changes in CLL tumor cells. Interestingly, the inhibition of glutamine metabolism in CD40/BCR-activated CLL cells abrogated resistance to venetoclax [24].

Another means of favoring tumor cell expansion involves the interaction between CLL cells and the TME and then the establishment of an immunosuppressive milieu [11]. For example, the presence of CLL cells is correlated with T cell exhaustion (by impeding glucose metabolism [25,26]) and T-cell dysfunction (via overexpression of the programmed death ligand (PD-L)1 in CLL cells). In CLL, macrophages display pro-tumor M2 differentiation, and the combination of impaired glucose metabolism and programmed death 1 (PD-1) activation results in monocyte dysfunction and a subsequent defect in immune surveillance [11].

Given the importance of the TME’s stimulation of CLL B cell survival and expansion, CLL-TME interactions have become therapeutic targets [27]. The TME’s signals include direct cell–cell and cell–extracellular matrix (ECM) contacts, the release of soluble factors (chemokines, interleukins, growth factors, and matrix metalloproteinase 9), and the release of small extracellular vesicles (EVs). A growing body of evidence shows that the EVs released by normal and tumor B cells are key components of the cancer-supporting TME [10,11]. Here, we review the biology of EVs in general, data from the current literature on the EVs’ expression profiles and roles in CLL, and the EVs’ putative functional value in countering drug resistance in CLL.

## 2. EV Generalities

EVs are small vesicles known to mediate intercellular communications in local and distant microenvironments under physiological and pathological conditions [28,29,30,31]. They carry a large variety of proteins, metabolites, DNA, RNA, microRNAs (miRNA), and long non-coding RNAs, and are secreted in biological fluids (urine, blood, ascites, and cerebrospinal fluid) [28,29,30,31].

Given the explosion of papers about EVs published in the last decade, the corresponding variety of protocols to purify them, and the continuous discovery of new vesicle types and characteristics, leading to the evolution of their classification, some guidelines have been edited by the International Society for Extracellular Vesicles (ISEV) for the clarification of EV nomenclature and protocols. The Minimal Information for Studies of Extracellular Vesicles (MISEV) guide was first published in 2014 [32], updated in 2018 [33], and is about to be updated again.

### 2.1. Nomenclature and Biogenesis

Based on MISEV 2018 [33], EVs are defined as nucleus-free particles with a bilayer lipid membrane, and they are released by cells in the extracellular space. EVs were initially separated into two main categories [28,31]:

- Exosomes (EXOs) are 30–150 nm vesicles generated through endosome maturation, the formation of multivesicular bodies (MVBs), intraluminal vesicles, and the fusion of MVBs with the plasma membrane. The secretion of EXOs is regulated by the endosomal sorting complexes required for transport (ESCRT) machinery. Therefore, some common EXO markers include ESCRT proteins such as Tsg101 and Alix. Tetraspanins CD9, CD81, and CD63 are also amongst the most popular EXO markers.
- Microvesicles (MVs) (previously referred to as ectosomes or microparticles) are 150–1000 nm vesicles resulting from the blebbing of the plasma membrane.

Despite intense research efforts developed to precisely describe the two different biogenesis pathways, there is still no consensus about the markers that can segregate these two populations, most likely because these processes are not exclusive. As some confusion has been seen over time in research articles regarding the nature, denomination, or origin of EVs, the ISEV was led to recommend the use of the generic name “extracellular vesicles”. When dealing with EV subtypes, a proposed alternative is to name EVs according to their size (small, medium/large EVs), density, surface expression markers, and/or any source/condition parameter that defines them [33]. With respect to research articles about EVs in CLL and other malignancies, most studies relate to EXOs, which are mainly defined as small-size vesicles isolated by ultracentrifugation. Independent of the EV’s nature, vesiculation can be modulated by different factors, including treatment with Ca^2+^ ionophores, temperature, pH, oncogenic transformation, cytoskeleton remodeling, or stress signals such as hypoxia [28].

### 2.2. Purification

EVs can be prepared from cell culture supernatants or biological fluids. Many different protocols have been developed. However, the gold standard remains purification by differential ultracentrifugation; that is, a series of centrifugations at different speeds to collect sequentially intact cells, dead cells and cell debris, then MVs, and finally EXOs [34]. An optimization of this protocol is density gradient ultracentrifugation, which improves the purity of collected EVs. Other techniques include polymer-based precipitation, ultrafiltration, size-exclusion chromatography, or immunoaffinity-based methods (ELISA and beads) [34]. Parameters that differ between these different techniques are purity, cost, time, yield, and specificity (the specific selection of EV markers or, on the contrary, a broad size selection). The choice of the optimal protocol also depends on the quantity of EV material available, the type of EVs, the volume of sample to analyze, and the application chosen [33].

### 2.3. Uptake of EVs and Transfer of Their Cargo to Recipient Cells

EV targeting to recipient cells can be mediated by cell surface receptors that specifically recognize EVs or through unspecific processes (such as micropinocytosis or macropinocytosis) [29,31,35]. While the fusion of EVs with the plasma membrane can result in the release of their content into the cytoplasm of recipient cells, EVs can also be internalized and fused with intracellular endosomes.

Following their internalization in recipient cells, the EV cargo can activate a variety of signaling pathways that regulate distinct biological functions in tumor cells related to proliferation, differentiation, migration, metabolism, drug resistance, or the survival/cell death balance [30]. Communication through EVs can therefore ultimately influence metastasis formation, immune escape, and a multitude of signals involving the TME.

## 3. CLL EVs and Their Role in the TME

CLL EVs have diagnostic and prognostic value in CLL. The secretion of EVs into the bloodstream contributes to the progression of CLL [10,36,37,38]. Thus, by targeting these EVs, one can reasonably expect to disrupt the CLL–TME interaction and increase the effectiveness of cancer treatments [39,40].

### 3.1. Purification and Characterization of CLL EVs

**Figure 1:**
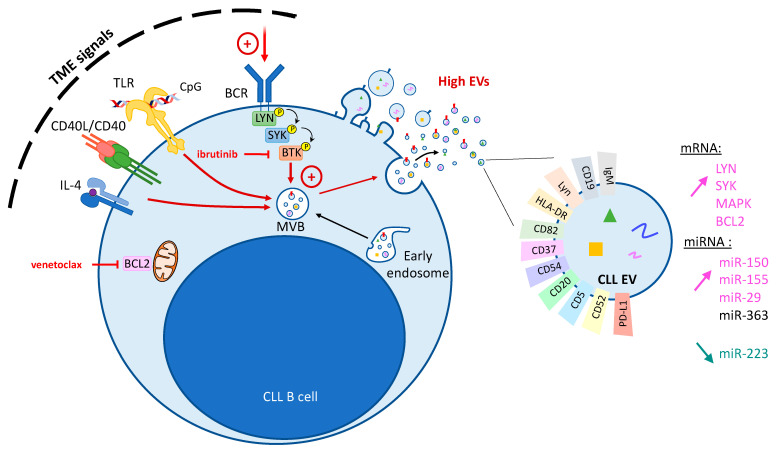
Modulation of EVs in CLL. The fusion of MVBs (multivesicular bodies) with plasma membrane or the budding of the plasma membrane leads to the release of CLL EVs from CLL B cells. EVs’ release can be increased by TME (tumor microenvironment) signals recapitulated by the stimulation of the BCR (B-cell receptor), TLR (Toll-like receptors) signaling, or CD40/IL-4 (left panel). CLL EVs are characterized by the exposure of specific surface markers (right panel). Compared to normal B cell EVs, CLL EVs contain a higher level of LYN, SYK, MAPK and BCL2 mRNAs, as well as a higher level of miR-150, miR-155, and miR-29 but a lower level of miR-223. TME stimulation increases their miR-363 content (BTK—Bruton’s tyrosine kinase; LYN—Lck/Yes novel tyrosine kinase; SYK—spleen tyrosine kinase).

Several research groups have compared and optimized various protocols for the purification and characterization of CLL EVs from cell culture supernatants or patients’ plasma [41,42,43]. Other than the conventional EV markers, several CLL-specific surface markers have been identified; these include components of the BCR pathway (such as IgM, CD19, and Lyn) and other molecules (such as HLA-DR, CD82, CD37, CD54, CD20, CD5, and CD52) [41,42,44,45,46,47,48,49] (Figure 1).

Here, we review the involvement of EVs (EXOs or MVs) in CLL biology in general. Technical details concerning the types of EV considered and the purification methods used in the research reviewed are summarized in Table 1. Conflicting results have been obtained by laboratories using different EV purification protocols, and techniques have evolved rapidly over the last few years.

### 3.2. Modulation of CLL Vesiculation by TME Signals

Despite their low proliferation rate in vitro, B cells from CLL patients spontaneously release MVs and EXOs in this setting. Furthermore, the plasma or serum levels of these EVs are higher in CLL patients than in healthy controls [45,47,48,50,62] (Table 1). Similarly, PB levels of EVs are abnormally high in patients with other hematological malignancies, including Waldenström’s macroglobulinemia, Hodgkin’s lymphoma, multiple myeloma, and acute myeloid leukemia [62].

EV release can be modulated by stimulating or blocking TME signals (Figure 1 and Table 1). For example, in vitro BCR stimulation with anti-IgM antibodies induces EXO release by CLL B cells; conversely, BCR inactivation with ibrutinib prevents this induction [47]. BCR ligation with anti-IgM treatment also increases CD52+ MV release by CLL B cells, although the total amount of MVs was not significantly influenced by ibrutinib treatment of CLL cells in vitro [45]. The treatment of CLL cells with idelalisib also blocks the EXO secretion resulting from anti-IgM stimulation [47], demonstrating that BTK/PI3K are involved in EXO release. Interestingly, the release of MVs in vitro and the release of plasma EXOs after BCR stimulation was greater in CLL cells from patients with a poor prognosis UM-*IGHV*, even though the difference vs. mutated-*IGHV* patients was not statistically significant [45,47].

Although it is clear that EXOs and MVs are released after BCR stimulation in vitro and in vivo, the data on inhibition by ibrutinib are contradictory. As observed in vitro, Yeh et al. found lower plasma EXO levels in CLL patients treated for one month with ibrutinib [47]. In the study by Boysen et al. of CLL patients, the plasma level of CD52+ MVs was low after three months of ibrutinib treatment; this might reflect a decrease in the total number of tumor cells after response to ibrutinib [45]. In the long term, however, an opposing trend was observed, with higher levels of CD52+ MVs in most patients [45]. This relative increase was not correlated with disease progression [45]. It is noteworthy that an electron microscopy analysis revealed that the purification method used by Boysen et al. resulted in a mixture of MVs and EXOs, which might explain the interstudy difference in profiles. More recently, the quantification of EXOs in a small cohort of ibrutinib-treated CLL patients did not evidence a significant decrease after 3 or more months of treatment [63]. However, it is important to note that several variables can influence EV release. Firstly, the latter three studies indicated changes over time in EV release: in all three cases, there was an initial decrease after ibrutinib treatment, despite some differences thereafter. Secondly, interindividual variability in small cohorts of CLL patients (*n* = 9, 5, and 14 in the studies by Yeh et al. [47], Boysen et al. [45], and Ishdorj et al. [63], respectively) would require a greater number of plasma samples for more precision. Most importantly, the studies differed in the selectivity of the technique used to quantify the EVs. EVs in the blood of ibrutinib-treated patients were variously measured in (i) a 100 k ultracentrifugation fraction, using NanoSight nanoparticle tracking [47], (ii) a 16 k fraction assayed for CD52 [45], and (iii) clarified supernatant assayed for CD9 [63]. These differences in purification and analysis techniques highlight the difficulty in comparing studies. Lastly, these interstudy differences probably also result partly from combinations of biological variabilities: the blood EV level might reflect not only BCR activation/inhibition but also the tumor cell count and levels of therapy-induced cell death. Nevertheless, the above-cited studies have clearly evidenced a link between BCR activation and EV release both in vitro and in vivo. Further investigation of the EVs’ involvement in anti-BCR therapy is therefore warranted.

The activation of other mediators of the CLL–TME interaction can also influence EV secretion (Figure 1). The stimulation of normal B cells’ interleukin (IL)-4 receptor and CD40 mimics activation by T cells and increases the release of EVs [64]. In CLL B cells, however, the CD40/IL-4 activation leads to a change in the EVs’ miRNA content but not in the EV count [51]. Treatment with CpG and thus stimulation of Toll-like receptor (TLR) signaling is associated with greater EV secretion by CLL cells; next-generation sequencing of the EVs’ contents revealed an enrichment in mRNAs related to BCR signaling [46].

It is noteworthy that the TME’s three inducers of EV release (i.e., BCR activation, CD40/IL-4 stimulation, and TLR stimulation) converge on the NF-κB pathway.

### 3.3. Influence of CLL EVs on the TME

**Figure 2:**
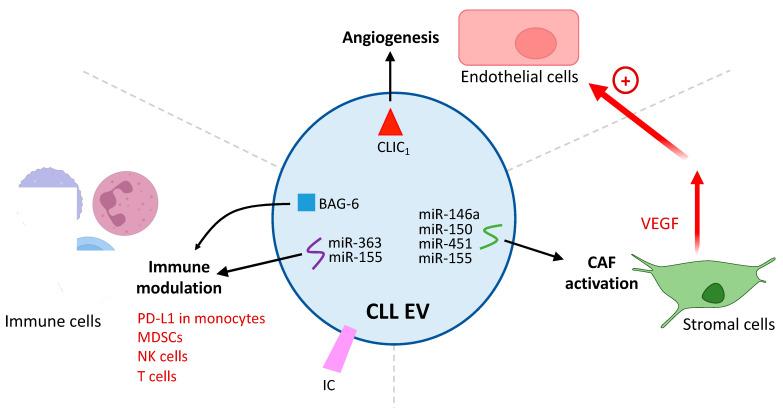
Influence of CLL EVs on TME. CLL EVs have the ability to influence the different cells comprising the TME through the delivery of their cargo to recipient cells, leading to a modulation of TME cell signaling in favor of the tumor. This includes the activation of stromal cells into CAFs (cancer-associated fibroblasts), increased angiogenesis in endothelial cells, and the modulation of the antitumor immune response (see text for details) (BAG6—BCL2-associated athanogene 6; CLIC1—chloride intracellular channel 1; IC—immunological checkpoint; MDSC—myeloid-derived suppressor cell; NK—natural killer; PD-L1—programmed death ligand 1; TME—tumor microenvironment; VEGF—vascular endothelial growth factor).

CLL–TME communication (whether mediated by direct contact or soluble molecules) is a two-way process [10,11,12]. When the TME influences the release of EVs by CLL cells (as detailed above), the CLL EVs can further modulate the TME (Figure 2). Indeed, EVs deliver their cargo to the various cell types in the TME and thus remotely alter cell signaling and (ultimately) tumor cell expansion and dissemination [36]. We describe three important examples of this below.

#### 3.3.1. Differentiation of Stromal Cells into CAFs

A transcriptomic analysis of stromal cells exposed to CLL EXOs containing miR-202-3p showed that the delivery of their contents increased the proliferation of stromal cells and the latter’s expression of c-fos and ataxia–telangiectasia mutated (ATM) [44]. Moreover, Paggetti et al. showed that CLL EXOs are internalized by BM stromal cells (MSCs and endothelial cells), which receive miRNAs (including miR-150, miR-155, miR-146a, and miR-451) and proteins that induce the inflammatory phenotype characteristic of CAFs [52] (Figure 2). Additional work demonstrated that CLL exosomal miR-146a induced the transition to CAFs via the upregulation of USP16 and the consequent induction of the CAF markers α-smooth muscle actin and fibroblast-activated protein, which drove tumor cell expansion [53].

This conversion of stromal cells into CAFs by EVs has been described in many tumor types. Conversely, once activated, CAFs can also secrete EVs that influence tumor progression. Through the transfer of a variety of molecules, including proteins, non-coding RNAs, and metabolites, CAF-EVs can influence the tumoral process at different levels by regulating the proliferation of tumor cells, their dissemination to form metastasis, and the antitumor immune response [65,66]. Importantly, CAF-derived EVs can also modulate the tumor response to therapy [67]. Hence, CLL EVs activate stromal cells and cause them to differentiate into CAFs. The EVs secreted by these activated cells then provide the CLL cells with survival signals, this vesiculation being dependent on LYN kinase [68]. MSC-derived EVs protect CLL cells from spontaneous apoptosis by inducing not only the expression of anti-apoptotic proteins but also cell migration, drug resistance, and BCR signaling activation [54]. Stromal cell EVs might therefore be the trigger in the established correlation between BCR activation and CLL EV vesicle release (see Section 3.2.). Importantly, CLL cell migration and gene expression were induced more strongly by EXOs from CLL patients’ MSCs than by EXOs from healthy patients’ MSCs [54]. One can reasonably hypothesize that CLL EVs prime stromal cells for activation, and this creates a regulatory loop through which stromal EVs accentuate the permissive microenvironment for CLL proliferation and drug resistance.

#### 3.3.2. Induction of a Pro-Angiogenic Phenotype

CLL EXOs increase the formation of blood vessels in vitro and in vivo [52]. In human umbilical vein endothelial cells, the transfer of chloride intracellular channel 1 (CLIC1) from CLL cell EVs is associated with greater proliferation and greater angiogenesis (Figure 2). This stimulation of angiogenesis involves the integrin-β1-dependent regulation of VEGF [55], which is a known pro-angiogenic survival factor in CLL [69]. Furthermore, CLL EXOs activate BM stromal cells by inducing HIF-1α signaling and thus VEGF production [50]. Interestingly, the induction of VEGF production was far more intense in CLL bone marrow stromal cells (BMSCs) than in BMSCs from healthy donors [50], suggesting that these cells had already differentiated into CAFs (mediated or not by EVs).

#### 3.3.3. Immunomodulation by CLL EVs

EVs delivered by CLL cells can influence immune cells and thus contribute to immune suppression and tumor immune escape (Figure 2). For example, the internalization of non-coding RNA from CLL EXOs induces the expression of cytokines and the immunosuppressant molecule PD-L1 in monocytes [56]. Very recent work has shown that CLL EXOs transmit endoplasmic reticulum stress to monocytes via extracellular nicotinamide phosphoribosyltransferase, which, in turn, promotes macrophage survival, a phenotype shift, and the secretion of inflammatory cytokines [57]. Similarly, the transfer of miR-155 from CLL EXOs induces the formation of immunosuppressive myeloid-derived suppressor cells; this process is inhibited by pretreatment of the CLL cells with vitamin D [58].

EVs purified from CLL cells can also impede antitumor immune surveillance by blocking the proliferation, activation, and metabolism of T lymphocytes and promoting T cell exhaustion and the formation of regulatory T cells [59]. Böttcher et al. suggested that these processes are regulated by the detection of immunological checkpoints (ICs) on CLL EVs, although a causal relationship with T cell dysregulation was not demonstrated. These results were confirmed and extended by a study of the Eμ-TCL1 CLL mouse model [60]. CLL EVs can reprogram the transcriptome, proteome, and metabolome of CD8+ T lymphocytes, causing exhaustion, an miRNA-dependent decrease in granzyme B, a fall in cytokine production, and tumor immune escape. Interestingly, Gargiulo et al. were able to correlate the elevated expression of genes involved in EV biology in CLL patients’ cells with the presence of markers of a poor prognosis and with poor survival [70].

However, EVs can sometimes activate an immune response. For example, the presence of BCL2-associated athanogene 6 (BAG6, a ligand of the NKp30 receptor in natural killer (NK) cells) in B cell EXOs can lead to NK activation and B cell lysis [71]. Conversely, soluble BAG6 induces the NK cells’ cytotoxic activity. A dysregulated, high soluble exosomal BAG6 ratio in CLL results in the impairment of NK cytotoxicity and thus promotes tumor immune escape.

As described in Section 3.2, vesicle release by CLL cells can be modulated by a variety of factors. Restoration of the T-cell/CLL cell interaction via CD40/IL-4 stimulation resulted in an enrichment in miR-363 in EXOs; this enrichment induced the downregulation of the immunomodulatory receptor CD69, increased T cell migration and proliferation, and elevated immune synapse signaling [51].

## 4. Clinical Implications of EV Biology in CLL

On the clinical level, EVs can be used as biomarkers of disease progression. Since CLL EVs participate in CLL progression at several levels by modulating cell proliferation and survival, cell migration, angiogenesis, treatment resistance, and immune escape, these vesicles are promising tools for (i) the improvement of current therapies for CLL and other hematological disorders, and (ii) the design of novel therapeutic strategies [37,72,73,74,75].

### 4.1. The EV Profile as a Biomarker in CLL

Although the circulating EV count is higher in CLL patients than in healthy controls, it is also clearly correlated with the Rai stage and thus serves as a marker of disease progression [48,50,62]. A high EV count is correlated with advanced disease and with other markers of a poor prognosis and poor survival; it therefore constitutes an independent prognostic factor [38].

Several groups have looked for (but failed to find) a relationship between CLL EVs and the lymphocyte count [47,48,51]. At first sight, it is tempting to think that the circulating EV count does not therefore reflect the tumor burden. In fact, the count might be a better marker of tumor cells because it takes into account the EVs released by CLL cells in the circulation and those resident in the BM and secondary lymphoid organs.

The analysis of EV surface markers in CLL has shown that some are biomarkers of disease progression. Advanced disease is correlated with elevated concentrations of MVs expressing CD19, CD20, and CD37 on their surface [48]. The accumulation of CD52^+^ MVs is also correlated with disease progression in treatment-naïve CLL patients [45].

Non-coding RNAs (including miRNAs) are crucial regulators of CLL progression and constitute valuable biomarkers of disease progression and treatment response [38,76,77]. The RNAs are packaged inside circulating EXOs and are thus protected from ribonuclease degradation. Moreover, the circulating EVs’ miRNA profile appears to be very different from that of the cells of origin and is therefore a very potent prognostic factor [38]. In CLL, the plasma EXOs’ specific miRNA profile includes the upregulation of miR-150, miR-155, and miR-29 family members and the downregulation of miR-223 [47]. The release of exosomal miR-150 and miR-155 was further elevated by anti-IgM treatment in vitro, which highlighted the correlation with BCR activation. MiR-155 in plasma EVs was shown to be correlated with CLL progression, survival, and treatment response [61,78]. Conversely, the transfer of miR-155 by EXOs has not been reported. However, the observation that miR-155 increases CLL cells’ responsiveness to BCR ligation/activation suggests that the BCR is transactivated by the EXO-mediated transfer of miR-155 between cells [79]. CD40/IL4 stimulation of CLL cells results in an enrichment of miR-363 in the CLL EVs [51]. It is noteworthy that the serum level of miR-150 is also a prognostic factor in CLL. However, most of the circulating miR-150 is free in plasma and not encapsulated in EVs [80]. In addition to miRNAs, the non-coding RNAs of interest in CLL include circular RNAs; for example, CLL EVs contain elevated levels of mc-COX2 (a mitochondrial genome-derived circular RNA associated with a poor prognosis) [81]. Aside from non-coding RNAs, mRNAs detected in CLL EVs also constitute great biomarkers, with a signature including some BCR specific kinases and apoptosis regulators (LYN, SYK, MAPK, and BCL2) [46].

Regarding the protein content of CLL EXOs, a large-scale proteomic analysis of samples from patients with indolent disease vs. progressive disease showed that the profile varied with disease progression [82]. In particular, exosomal S100 calcium-binding protein A9 (S100-A9) was identified as a marker of disease progression and an activator of the nuclear factor-kB pathway in CLL cells. CLL EVs also expressed immune checkpoint ligands (including PD-L1) at their surface [60].

A small proportion of the CLL patients who become refractory to chemotherapy will go on to develop a more aggressive lymphoma known as Richter syndrome (RS) [83,84]. Although epigenetic modifications have been described as CLL progresses into RS, the underlying mechanisms are still not completely understood, and biomarkers of this transformation are needed. In a proof-of-concept study, Jurj et al. identified exosomal miR-19b as a regulator of CLL cell proliferation and invasion and thus as a predictive biomarker of RS transformation [85].

In summary, EVs appear to be important, relatively non-invasive diagnostic, prognostic, and disease progression biomarkers in CLL.

### 4.2. Interference with Immunotherapy

Given the great variety of cargo molecules disseminated into the TME, CLL EVs not only contribute to disease progression but also hamper the treatment of CLL.

Two recent studies of anti-CD19 chimeric antigen receptor (CAR)-T cell therapy showed that CLL EVs can disrupt the immune synapse and contribute to the CAR-T-cell’s exhaustion, metabolic quiescence [59], or even lysis [86]. These findings might explain the CAR-T-cell treatment failure observed in the clinic. The researchers also demonstrated the presence of several different ICs (including PD-L1) on the surface of CLL EVs and suggested that this might explain the failure of CAR-T-cell therapy and anti-PD-1/PD-L1 therapy in CLL. The various ICs detected on EVs might compensate for each other when a single one is targeted. Furthermore, the exposure of PD-L1 on the EV surface might compete for anti-PD-L1 antibodies with cell-surface PD-L1.

Similarly, the surface presentation of B cell surface markers on EVs can interfere with immunotherapy. Neutralization of the anti-CD20 antibody rituximab by CD20 exposed on the surface of B-cell lymphoma EXOs leads to immune evasion [52,87,88], and the blockade of ABCA3-regulated vesiculation increased the effectiveness of rituximab therapy [87]. A similar scenario might apply to other B-cell membrane antigens bound to EXOs, such as CD19, CD37, and HLA-DR [88].

### 4.3. Novel EV-Driven Therapeutic Strategies

Despite of their potential interference with CLL immunotherapy, EVs might also constitute a valuable curative treatment for CLL [37,74,75].

As mentioned above, exosomal BAG6 and soluble BAG6 have opposing effects on NK cell activation and antitumor immunity; hence, treatment with BAG6-containing EXOs might restore the NK cell’s ability to kill CLL cells [71]. EVs are now being considered as new therapeutic vehicles, with the custom delivery of drugs or molecules packaged in engineered EVs [37]. Thus, several studies have exploited the natural affinity of Epstein–Barr virus for B cells by developing custom EXOs that specifically target CLL cells. In one study, CLL B-cells were specifically targeted by EXOs tagged with the viral envelope protein gp350, leading to the exosomal co-transfer of gp350 and CD40L to the patients’ cells and thus the stimulation of an anti-CLL T-cell immune response [89]. In a second study, gp350-labelled EXOs transferred both CD40L and pp65 protein to CLL cells and thus activated a T-cell immune response [90]. Very recently, the same principle was used to deliver fludarabine specifically to CLL B cells via gp350+ EVs purified from engineered red blood cells [91].

On the other hand, given the EVs’ crucial role in CLL progression (as described above), targeting CLL vesicles (by blocking vesiculation in donor cells or uptake by recipient cells) might block tumor–TME communication [37]. For example, the inhibition of vesiculation through the targeting of Rab27a in B cells improves the post-chemotherapy antitumor response [92]. On the other hand, the pretreatment of CLL EVs with low-molecular-weight heparin, a heparan sulfate analog, blocks the uptake of EVs by stromal cells [52]. Interestingly the authors noticed that CLL EVs were not internalized by CLL cells, and they suggested this could be linked to the difference in the exposure of heparan sulfate proteoglycans at the surface of the cells.

However, one major limitation might be a lack of specificity because the molecular drivers of vesiculation are common to many physiological processes. Hence, CLL-EV-specific mechanisms must be discovered, or EV inhibitors must be specifically delivered to CLL cells. For example, the CLL-specific delivery of Rab27a siRNA was performed by Zhang et al. through inactivated Epstein–Barr virus, limiting the inhibition of vesiculation to B cells [92]. One can also imagine that therapeutic EVs containing vesiculation inhibitors could be optimized for the delivery to CLL cells through the expression of specific tumor surface proteins and/or their production in mesenchymal stem cells that display a natural tropism toward tumor cells.

Overall, using EV communication to develop novel therapeutic strategies or to optimize current CLL treatments definitively hold some promise, but several challenges still remain [75]. To be suitable for clinics, EV-based therapies will need high-throughput technologies to produce and purify large quantities of standardized EV preparations with no off-target effects. Therapies targeting tumor EVs will also be challenging because of the shared mechanisms between physiologic and tumor vesiculation. Nevertheless, EV research has developed very rapidly during the last decade, and the numerous ongoing research works on tumor EVs will allow for the further elucidation of the modalities of EV secretion and uptake to design innovative therapeutic strategies for CLL and other tumor types.

## 5. Conclusions

Over the last 10 years, a large number of scientific articles on CLL EVs have been published. Several important points stand out. Firstly, the intensity of EV release and the nature of the EV contents might serve as biomarkers of CLL progression and the treatment response. Circulating EVs are particularly relevant diagnostic and prognostic biomarkers of solid tumors because a blood sample is less invasive than a tissue biopsy. However, circulating EVs are still of interest for the diagnosis and prognosis of B-cell malignancies such as CLL [37,38]. In CLL, the main prognostic factors are clinical observations, the *IGHV* mutation status, and genetic abnormalities. The EV count and the nature of the EVs’ content (particularly miR-155) are correlated with CLL progression, BCR activation, and overall activation by the TME (see Section 3.2. and Section 4.1.) and thus constitute valuable biomarkers for CLL.

Secondly, EVs are actively involved in the dialog between CLL cells and the TME, which, in turn, provides the tumor cells with immune protection and a survival advantage (see Section 3.3.). The blockade of CLL EV trafficking would constitute a novel treatment option or an adjunct to current treatments for CLL.

Lastly, the rapid development of EV engineering technologies for the treatment of cancer suggests that novel, targeted approaches are possible, such as the EV encapsulation of CLL drugs and the expression of CLL-specific proteins by EVs [37,75]. It is important to bear in mind that the presence on CLL EVs of molecules that are also expressed on the surface of CLL B cells may impact CLL therapy. On one hand, this might be to the patient’s disadvantage; for example, exosomal CD20 and PD-L1 neutralize anti-CD20 therapy [87] and CD19-CAR-T cells [86], respectively. More generally, the presentation of ICs at the EV surface impedes an antitumor immune response [60]. On the other hand, it should be possible to engineer EVs to express molecules also present on the surface of TME cells; the resulting competition process might inhibit tumor–TME communication and thus impede disease progression.

Several CAR-T agents (including anti-CD19 CAR-T (lisocabtagene), anti-CD20 CAR-T (C-CAR066), anti-CD19/CD20 CAR-T, and anti-CD20/CD22 CAR-T) are being tested as treatments for rituximab-resistant CLL in combination with other chemotherapeutics [6]. However, the observation of unexpected negative results and adverse events raised the question of whether CAR-T cells could be replaced by the CAR-T-EVs they generate. Very interestingly, a recent study showed that CAR-T-cell-derived EXOs carried the CAR but also had their own cytolytic activity, which led to the inhibition of tumor growth in two distinct mouse models [93]. In contrast to CAR-T therapy, CAR-T-EV therapy does not appear to be inhibited by the PD-1 pathway or induce cytokine release syndrome; it might therefore constitute a safer treatment option. Although further development is clearly needed, CD19-CAR-T-EVs have already been used to target B-cells in acute lymphocytic leukemia [94]; the transfer of this technology to CLL would be of great interest.

In summary, when considering the large number of activities exerted by CLL EVs and their roles in TME cross-talk, treatment response, and the development of resistance, targeting these vesicles might open up new therapeutic approaches. Complementary research is needed, but the combination of EV-based approaches with the currently available CLL therapies could undoubtedly help optimize treatment outcomes and improve patients’ quality of life.

## Figures and Tables

**Table 1:** Summary of discoveries and protocols used in the main articles about CLL EVs discussed in the review (UC—ultracentrifugation; CLL cell lines: EHEB—HG3—MEC-1—PGA-1; endothelial cell lines: HMEC-1—HUVEC; human BM cell lines: HS5; monocytic cell line: THP-1; primary fibroblasts: HDFn; ↑: increase; ↓: decrease).

Donor Cells	EVs	EV Markers	Cargo	Purification Protocol	Target Cells	Results	Ref
Untreated CLL patients (*n* = 34) and healthy donors, MEC-1	EXO	TSG101, MHC I/II, IgM, Lyn kinase, CD81, CD37, ITGA4	miR-202-3p	10 min at 500× *g*, 10 min at 4000× *g*, 30 min at 18,000× *g*, filtration (Immuno-magnetic isolation), UC 90 min at 100,000× *g* (×2)	HS-5	CLL EXOs ↑ expression of genes such as c-fos and ATM; ↑ proliferation of recipient HS-5 cells; CLL EXOs enriched in certain miRNAs	[44]
Primary CLL B-cells and normal B cells, plasma (*n* = 33 CLL/*n* = 9 treatment-naïve CLL patients/*n* = 5 CLL patients under ibrutinib)	MV	CD52	-	20 min at 2500× *g* (×3); 1 h at 16,000× *g* at 4 °C	-	↑ CD52+ MVs with BCR stimulation in CLL B-cells; ↑ plasma CD52+ MVs correlated to tumor progression; ↓ plasma CD52+ MVs after ibrutinib therapy	[45]
Primary CLL and healthy B cells	EV	CD63, CD9, CD54, CD82	TCL1A-mRNA	10 min at 300× *g*, 15 min at 6800× *g* (×2), UC 90 min at 100,000× *g* (×2)	HDFn and THP-1	CLL-CpG-EVs contain disease-relevant mRNA; ↑ CLL-EVs compared to healthy B cells	[46]
CD19+ B cells from CLL patients and healthy donors	EXO	CD63, CD9, CD37	miR-155, miR-150	10 min at 300× *g*, 10 min at 2000× *g*, 30 min at 10,000× *g* at 4 °C, UC 70 min at 100,000× *g* at 4 °C (×2)	-	↑ EXOs in CLL patients’ plasma; ↑ EXOs with BCR activation by α-IgM in CLL B cells; ibrutinib impedes α-IgM-stimulation EXO release; ↑ EXO miR-150 and miR-155 with BCR activation	[47]
Serum (*n* = 131 CLL/*n* = 28 healthy controls)	MV	CD19, CD37		20 min at 2000× *g* at 4 °C, 30 min at 10,000× *g* at 4 °C, UC 70 min at 100,000× *g* at 4 °C (×2)	-	↑ MVs in CLL patients’ plasma; CD19+ CD37+ MVs correlate to tumor progression; total MVs predict for overall survival and time to treatment	[48]
Plasma (*n* = 60 CLL, *n* = 5 healthy controls)	MV	CD19		20 min at 2500× *g* (×3), 1 h at 16,000× *g* at 4 °C	HS-5, primary BMSCs	↑ MVs in CLL patients’ plasma; ↑ VEGF, B-catenin pathway, cyclin D1, and c-myc in CLL-BMSCs	[50]
CD19+ CD5+ B cells CLL patient and healthy donors; plasma CLL patients	EV comprise EXO	-	miR-363, miR-155, miR-374b	5 min at 250× *g* at 4 °C, 10 min at 2000× *g* at 4 °C, 30 min at 10,000× *g* at 4 °C, UC 110 min at 100,000× *g*	CD4+ T cellsfrom CLL patients	CD40/IL-4–stimulated CLL cells released specific EV miRNAs; ↑ migration; proliferation of CD4+ T cells; immunological synapse signaling	[51]
Primary CLL (*n* = 21), MEC-1	EXO	ALIX, TSG101, HLA-DR		10 min at 400× *g* (×2), 20 min at 2000× *g*, filtration 0.45 μm, UC at 110,000× *g* at 4 °C, flotation on Optiprep cushion (Axis-Shield, 17%) for 75 min at 100,000× *g* at 4 °C, filtration 0.45 μm	Human BM-MSCs, HMEC-1, HS5	CLL-EXO transfer protein and miRNA into stromal cells that induce a CAF-like phenotype; uptake by endothelial cells ↑ angiogenesis	[52]
MEC-1	EXO	CD63, CD9	miR-146a	10 min at 400× *g* (×2), 20 min at 2000× *g*, filtration 0.45 μm, UC 70 min at 110,000× *g* (×2), 75 min at 100,000× *g* at 4 °C, filtration 0.45 μm	Human BM-MSCs	CLL cells deliver miR-146a to BM-MSCs that induce CAFs phenotype by down-regulation of USP16 mRNA expression	[53]
Human BM-MSCs	EV	CD63		10 min at 300× *g* (×2), concentrated on 3 K centrifugal device, UC 1 h at 150,000× *g* at 4 °C (×2)	Primary CLL	BM-MSCs ↓ B CLL spontaneous apoptosis and ↑ chemoresistance to fludarabine, ibrutinib, idelalisib and venetoclax; ↑ CLL B cells migration	[54]
Plasma CLL patients, MEC-1	EXO	CD63, CD81, TSG101	CLIC1	20 min at 400× *g*, 40 min at 2000× *g*, filtration 0,45 μm, UC 70 min at 110,000× *g* at 4 °C, floatation on Optiprep cushion (Axis-Shield, 17%) 75 min at 100,000× *g* at 4 °C, filtration 0.45 μm	HUVECs	MEC-1 EXO invasion; metastasis and angiogenesis of HUVECs by transferring CLIC1	[55]
Plasma CLL patients and healthy donors, MEC-1	EXO	RAB5a, HSP70, HLA-DR, CD81	noncoding Y RNA hY4	300× *g* and 10,000× *g*, UC at 100,000× *g*, UC on 40% sucrose cushion	Human monocytes or murine BM-MDSCs	↑ release of cytokines, such as CCL2, CCL4, and interleukin-6,and expression of PD-L1	[56]
Untreated CLL patients (*n* = 26, aggressive /indolent)and healthy donors, MEC1 and HG3	EXO	CD63, CD81	NAMPT	15 min at 3000× *g*, ExoQuick -TC reagent overnight at 4 °C, 30 min at 1500× *g*	Primary monocytes (CD14+ CD16+)	CLL-EXO transfer NAMPT to monocytes; ↑ NAD+ (nicotinamide adenine dinucleotide) which activatedSIRT1-C/EBPβ signaling pathway in monocytes	[57]
CLL patients (*n* = 56) and healthy donors, EHEB and MEC1, serum CLL patients and healthy donors	EXO	CD63, CD81		10 min at 500× *g*, 20 min at 16,500× *g*, filtration 0.2 μm, UC 70 min at 110,000× *g* at 4 °C (×2) and filtration 0.2 μm	CD14+HLA-Drlow monocytes (MDSCs)	miR-155 in CLL-EXO induces MDSCs; is disrupted by vitamin D	[58]
PB and plasma samples, CLL patient and healthy donors, MEC-1, HG-3, EHEB, and PGA1	EV	CD9, CD63, CD81, CD19, CD20, CD40	ICs	filtration 0.2 μm, UC 70 min at 110,000× *g* at 4 °C	T-cells	CLL-EV contain ICs that may hamper T-cell viability, proliferation, activation, and metabolism	[59]
Eμ-TCL1 CLL murine model (WT B cells from C57BL/6 mouse)	sEV (EXO)	Alix, TSG101, CD63, CD9, CD81	miR-150, -155, -21, -146a, -378a, and -27a, IC ligands	5 min at 400× *g*, 20 min at 400× *g*, 40 min at 2000× *g*, 60 min at 10,000× *g*, filtration 0.2 μm, UC 70 min at 110,000× *g* at 4 °C, flotation on 17% Optiprep cushion, 75 min at 100,000× *g* at 4 °C, UC 70 min at 110,000× *g* at 4 °C, filtration 0.45 μm and 0.22 μm	CD8+ T cells	small EVs secreted by CLL cells in mouse model inhibit CD8+ T-cell immune response against tumor cells	[60]
Serum (*n* = 9 CLL/*n* = 18 healthy controls)	EV			-	-	↑ miR-155 in CLL EVs	[61]

## Abbreviations

APRIL—a proliferation-inducing ligand; BAFF—B-cell activating factor; BAG6—BCL2-associated athanogene 6; BCL2—B-cell lymphoma/leukemia 2; BCR—B cell receptor; BM—bone marrow; BMSC—bone marrow stromal cell; BTK—Bruton’s tyrosine kinase; CAF—cancer-associated fibroblast; CAR-T cell—chimeric antigen receptor T cell; CD40L—CD40 ligand; CLL—chronic lymphocytic leukemia; ECM—extracellular matrix; ESCRT—endosomal sorting complexes required for transport; EV—extracellular vesicle; EXO—exosome; IC—immunological checkpoint; IGHV—immunoglobulin heavy chain region; ISEV—international society for extracellular vesicles; LN—lymph node; LYN—Lck/Yes novel tyrosine kinase; miRNA—microRNA; MISEV—minimal information for studies of extracellular vesicles; MSC—mesenchymal stromal cell; MV—microvesicle; MVB—multivesicular body; NF-κB—nuclear factor kappa B; NK—natural killer; PB—peripheral blood; PD-L1—programmed death ligand 1; PD-1—programmed death 1; PI3K—phosphoinositide 3-kinase; RS—Richter syndrome; SDF-1—stromal cell-derived factor 1; SYK—spleen tyrosine kinase; TLR—toll-like receptor; TME—tumor microenvironment; VEGF—vascular endothelial growth factor.
